# Mode of delivery and maternal vitamin D deficiency: an optimized intelligent Bayesian network algorithm analysis of a stratified randomized controlled field trial

**DOI:** 10.1038/s41598-023-35838-6

**Published:** 2023-05-29

**Authors:** Mina Amiri, Maryam Rostami, Ali Sheidaei, Aida Fallahzadeh, Fahimeh Ramezani Tehrani

**Affiliations:** 1 Reproductive Endocrinology Research Center, Research Institute for Endocrine Sciences, Shahid Beheshti University of Medical Sciences, Tehran, Iran; 2 Department of Social Medicine, Faculty of Medicine, Ahvaz Jundishapur University of Medical Sciences, Ahvaz, Iran; 3 School of Public Health, Department of Epidemiology and Biostatistics, Tehran University of Medical Sciences, Tehran, Iran; 4 School of Medicine, Tehran University of Medical Science, Tehran, Iran; 5 Reproductive Endocrinology Research Center, Research Institute for Endocrine Sciences, Shahid Beheshti University of Medical Sciences, 24 Arabi, Yaman Street, Velenjak, Tehran, 1985717413 Islamic Republic of Iran

**Keywords:** Endocrinology, Health care, Medical research

## Abstract

This study aimed to elucidate the algorithm of various influential factors relating to the association between 25-hydroxyvitamin D (25(OH)D) concentration at delivery and mode of delivery. The investigation constituted a secondary analysis using data collected as part of the Khuzestan Vitamin D Deficiency Screening Program in Pregnancy, which is a stratified randomized vitamin D supplementation-controlled trial comprising 1649 eligible pregnant women. The Bayesian Network (BN) method was utilized to determine the association algorithm between diverse influential factors associated with maternal vitamin D and mode of delivery. The optimized intelligent BN algorithm revealed that women presenting with moderate (35.67%; 95% CI: 33.36–37.96) and severe vitamin D deficiency (47.22%; 95% CI: 44.81–49.63) at delivery were more likely to undergo cesarean section than those presenting with normal concentrations of this nutritional hormone (18.62%; 95% CI: 16.74–20.5). The occurrence probabilities of preeclampsia in mothers with normal, moderate, and severe vitamin D deficiency at delivery were (1.5%; 95% CI: 0.92–2.09), (14.01%; 95% CI: 12.33–15.68), and (26.81%; 95% CI: 24.67–28.95), respectively. Additionally, mothers with moderate (11.81%; 95% CI: 10.25–13.36) and severe (27.86%; 95% CI: 25.69–30.02) vitamin D deficiency exhibited a higher probability of preterm delivery in comparison to those presenting with normal concentrations (1.12%; 95% CI: 0.62–1.63). This study demonstrated that the vitamin D status of pregnant women at delivery could directly affect the mode of delivery and indirectly through maternal complications, such as preeclampsia and preterm delivery, leading to a higher occurrence probability of cesarean section.

## Introduction

Vitamin D is recognized as a crucial nutrient, that plays a pivotal role in various physiological processes such as cell function, bone mineralization, calcium homeostasis, and muscle performance^1–3^. However, epidemiologic studies have demonstrated that pregnant women are at a higher risk of vitamin D deficiency^4–6^. A systematic review estimates that the prevalence of vitamin D deficiency, defined as serum 25(OH)D < 20 ng/ mL (50 nmol/L), ranges from 64 to 83% depending on geographic region^7^.

In addition to the role of vitamin D in maintaining calcium homeostasis and bone metabolism, a deficiency in vitamin D during pregnancy can result in various complications such as preeclampsia, gestational diabetes mellitus (GDM), and preterm delivery^8–11^, and an increased risk of cesarean Sect. ^12–17^. The mechanisms behind the association between 25(OH)D and increased cesarean section risk are complex and not fully elucidated. Vitamin D is important for smooth muscle performance and myometrium contractility which plays a critical role in the initiation of labor^18^. The deficiency of vitamin D can also lead to endothelial dysfunction, impaired vascular health^19^, and glucose homeostasis abnormalities^20,21^, which can predispose to developing GDM^22^. The levels of vitamin D can also affect preterm delivery through inflammation and immune system modulation. Additionally, vitamin D deficiency can increase the risk of infection, which is known to be a major cause of preterm delivery^10^. Maternal complications such as preeclampsia, GDM, and preterm delivery can indirectly lead to cesarean section due to fetal distress^23–25^. Studies suggest that 25(OH)D levels during delivery may influence the rate of cesarean and operative deliveries by increasing birth weight^26^, independently of gestational age^27^. Birth weight is linked to GDM, which can increase the risk of cesarean section due to cephalopelvic disproportion (CPD) and prolonged labor (dystocia)^28^.

Several studies have examined the relationship between 25(OH)D concentrations and mode of delivery, but their findings have been inconsistent and inconclusive^12–17,29–32^. Some studies have suggested that mothers with vitamin D deficiency are at an increased risk of cesarean Sect.^12–17^, while others have found no link between serum levels of 25(OH) D and type of delivery^29–32^. This discrepancy may be due to various confounding factors that were not accounted for in most of these studies.

To better understand and simplify the complex association between 25(OH)D concentration at delivery and mode of delivery, we utilized data from a stratified randomized controlled field trial of vitamin D supplementation. We employed a Bayesian Network (BN), which is a popular data mining algorithm to predict the likelihood of cesarean section in mothers with vitamin D deficiency. This innovative approach allowed us to elucidate the relationships among the variables in our analysis.

## Methods

### Study design and participants

This study presents a secondary analysis of data collected from the Khuzestan Vitamin D Deficiency Screening Program in Pregnancy, which was a stratified randomized controlled field trial. The study protocol and procedures have been previously published^33,34^.

This study was conducted in two phases. The first phase involved a population-based cross-sectional study that recruited 1,600 and 900 first-trimester pregnant women from health centers in Masjed-Soleyman and Shushtar, respectively. The sampling method used was stratified multistage cluster sampling with probability proportional to size (PPS). Inclusion criteria include age range 18–40 years, gestational age < 14 weeks, singleton pregnancy, not consuming multivitamins containing 400 IU/d of D3, and no previous history of chronic diseases. Participants consuming multivitamins containing more than 400 international units (IU) per day of vitamin D3 and anticonvulsants and those with a history of chronic diseases like diabetes, hypertension, renal dysfunction, liver diseases, and complicated medical or obstetrical history were excluded from the study. The study data was collected from 1800 first-trimester pregnant women referred to health centers in Masjed-Soleyman (intervention) and Shushtar (control), from whom fasting blood samples were collected. Serum samples of participants from Shushtar were stored and kept frozen at -80° C until further assays at the end of the study, according to the original study protocol. The vitamin D status of participants from Masjed-Soleyman was measured immediately, and those found to have vitamin D deficiency were randomly assigned to a treatment regimen consisting of vitamin D3 supplementation until delivery. During the second phase of the study, based on 25(OH)D levels, mothers were divided into severe deficient (< 10 ng/ml), moderate deficient (10-20 ng/ml), and normal status (> 20 ng/ml) (6). A total of 800 pregnant women with vitamin D deficiency (defined as serum concentrations of 25(OH)D < 20 ng/ml) from Masjed-Soleyman were included in the intervention group and received vitamin D supplementation according to the assigned protocol. Participants were randomized into groups of four using a computer-generated list. The physicians involved in the study were not informed about which group each woman was placed in, but the midwife who was not involved in any phase of the study knew. It was not possible to hide the treatment allocation from healthcare workers, but those who assessed pregnancy outcomes were unaware of the treatment allocation. The remaining women with vitamin D deficiency were referred to specialists for further treatment. Participants from Shushtar served as the control group and did not receive any vitamin D supplementation. Women with severe and moderate vitamin D deficiency from Masjed-Soleyman were randomly allocated to one of four treatment modalities: A1 (treatment with 50,000 IU of oral vitamin D3 weekly for a total duration of 12 weeks), A2 (treatment with 50,000 IU of oral vitamin D3 weekly for a total duration of 12 weeks and then on a monthly maintenance dose of 50,000 IU vitamin D3 until delivery), A3 (intramuscular administration of 300,000 IU vitD3; 2 doses for 6 weeks), and A4 (Intramuscular administration of 300,000 IU vitamin D3; 2 doses for 6 weeks and then on a monthly maintenance dose of 50,000 IU vitamin D3 until delivery). Women with moderate vitamin D deficiency were assigned to one of four study groups: B1 (treatment with 50,000 IU oral vitamin D3 weekly for a total duration of 6 weeks), B2 (treatment with 50,000 IU oral vitamin D3 weekly for a total duration of 6 weeks and then on a monthly maintenance dose of 50,000 IU vitamin D3 until delivery), B3 (a single dose of intramuscular administration of 300,000 IU vitamin D3), and B4 (a single dose of Intramuscular administration of 300,000 IU vitamin D3 and then on a monthly maintenance dose of 50,000 IU vitamin D3 until delivery). Serum levels of 25 (OH)D of all participants (from both cities) were measured at delivery. All participants (intervention and control groups) received routine prenatal care and followed till delivery.

**Figure 1:**
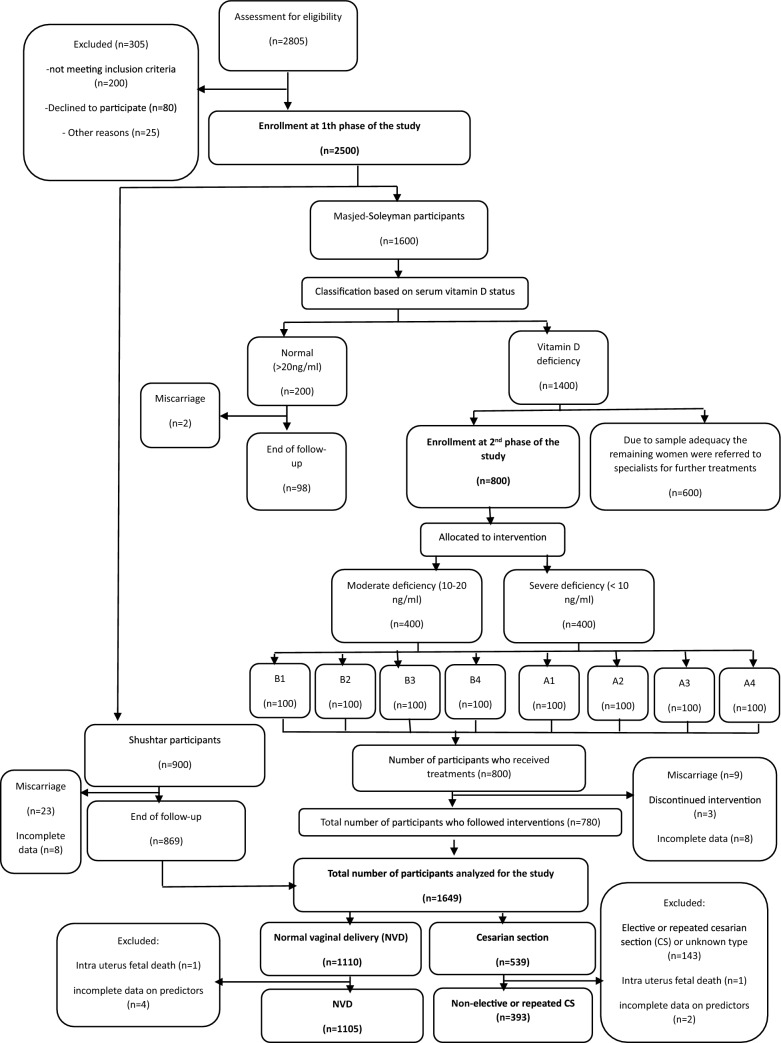
The study flow-diagram.

For this analysis, elective and repeat cesarean section cases, those without reliable data about the type of cesarean section (n = 143), IUFD (n = 2), and incomplete data on predictors (n = 6) were excluded. As a result, a total of 1488 pregnant women were included in the analysis (Fig. 1).

### Clinical and laboratory measurements

Clinical and anthropometric parameters were measured for all participants (by trained examiners at baseline and delivery. All participants were interviewed for sociodemographics, their history of pregnancies, and medical, obstetrics, and family histories using pretested questionnaires. Adverse pregnancy outcomes were defined based on the standard diagnostic criteria. At the time of data collection, women were asked about their history of preeclampsia, based on a self-reporting questionnaire at each follow-up, details of which have been previously published^33^. In brief, weight was measured with minimum clothing to the nearest 100 g. Height was measured with a tape measure in a standing position with a normal posture of the shoulders. Body mass index was calculated by dividing weight (kg) by height (m2). Systolic and diastolic blood pressures (SBP and DBP) were measured twice in a sitting position with a standard mercury sphygmomanometer after a 15-min rest, and the mean of the two measurements was considered as SBP or DBP. Serum concentrations of 25(OH)D were assayed for all participants (both Masjed-Soleyman and Shushtar) at baseline and at delivery using the ELISA method and a kit of Immunodiagnostics Systems by AutoAnalyzer (Human Corporation, Germany). The interassay and intraassay coefficients of variation were 3.891% and 3.37%, respectively (sensitivity of 5 nmol/L). The instruments were calibrated as per the manufacturer's instructions, and validation studies were performed before the test. Samples were analyzed by a single technician using the same equipment throughout the study in a reference laboratory and were measured according to standard operating procedures.

### Term definition

Preeclampsia was defined as having systolic blood pressure > 140 mmHg or diastolic blood pressure 90 mmHg and 24-h proteinuria 0.3 g, initiated at > 20 weeks of gestation^35^, GDM as glucose intolerance first detected during pregnancy, based on the criteria of the International Association of Diabetes and Pregnancy Study Groups^36^, and preterm delivery as birth at < 37 weeks of gestation^37^. Women with concentrations of 25(OH)D between 10 and 20 ng/mL diagnosed as moderate vitamin D deficiency and those with concentrations < 10 ng/mL considered as severe vitamin D deficiency.

### Study outcomes

The study outcome was the mode of delivery which was classified as either vaginal or emergency cesarean section. According to the ICD-10 classification system, an emergency cesarean section included all cases where such delivery was undertaken after the onset of labor. Dystocia refers to prolonged or slowly progressing labor and is defined as abnormal or difficult labor and may be associated with factors of the passenger (fetus), the passage (maternal pelvis like CPD), the forces (powers), or a combination of these factors. CPD refers to the inability of the fetus to pass through the pelvis.

### Statistical analysis

The available data appears to be appropriate for achieving research goals; however, some methodological considerations are necessary to address the complexity of the design. To simplify the relationships between variables, we used the BN method, one of the most well-known algorithms in data mining.

The statistical method employed to evaluate the generalizability of our findings was a tenfold cross-validation technique. The dataset was randomly divided into ten equal parts, with nine parts being utilized for model training in each iteration. The remaining part was used for evaluating the predicted values. To obtain a comprehensive performance evaluation, we calculated the sensitivity, specificity, and accuracy metrics by averaging these values across all ten iterations.

We employed the Hill-Climbing (HC) algorithm to achieve the best Directed Acyclic Graph (DAG) corresponding to our data. HC is a score-based structure learning algorithm that progressively restricts the neighborhood. Compared with constraint-based algorithms, score-based algorithms result in entirely directed DAG, which is their primary advantage^38^.

To fit the parameters of BN corresponding to DAG achieved in structure learning, we used the Bayesian estimation^39^. We predicted the type of delivery for individuals in the test set and compared it with the actual observations. To evaluate the validity of predictions, we used the Receiver Operating Characteristic (ROC) curve and the Area Under Curve (AUC). The conditional probability tables for the primary exposure and outcome variables were derived from the BN, and the chi-squared statistics were used to test the significance of relations.

Finally, we fitted several logistic regressions adjusted for potential confounders as the alternative approach for modeling the data. We compared the prediction power of these models with the suggested BN using the AUC of these models in the test datasets.

The statistical analysis, data preparation, and graphical representation were conducted using the R statistical software environment. We used the "bnlearn" package to fit the BN model^40^. The type one error for statistical inferences was defined at 0.05.

### Ethical approval and consent to participate

This research project was approved by the Medical Ethics Committee of the Research Institute for Endocrine Sciences (IR.SBMU.ENDOCRINE.REC.1400.042). All methods were performed in accordance with the relevant guidelines and regulations. All study participants provided written informed consent.

## Results

Table 1 summarized the characteristics of the study participants in the intervention and control cities. There was no significant difference in the education level, job status, number of children, and age at current pregnancy between women from the two cities. However, the other characteristics, such as the prevalence of pregnancy complications like GDM (3.8% in the intervention and 6.2% in the control city with *P*-value = 0.03), preeclampsia (8.9% in the intervention and 17% in the control city with *P*-value < 0.001), low birth weight (5.8% in the intervention and 11% in control city with *P*-value < 0.001), and preterm (9% in the intervention and 15% in control city with *P*-value < 0.001) were significantly higher in the control city than in the intervention city. The prevalence of moderate and severe vitamin D deficiency at baseline was 46% and 38%, respectively, in the control city, while in the intervention city, these values were 44% and 45%. Consequently, there was a significantly higher prevalence of severe vitamin D deficiency in the intervention city (*P*-value = 0.004). Severe vitamin D deficiency prevalence at delivery was reported at 1.5% and 45% in the intervention and control cities (*P*-value < 0.001), respectively. The prevalence of cesarean section in the case and control groups were 29% and 37%, respectively (*P*-value < 0.001).

**Table 1:** Characteristics of females who participated in the study and their husbands.

Characteristic	Overall, N = 1 649^1^	Masjed–Soleyman (Intervention) N = 841^1^	Shushtar (Control) N = 808^1^	*P* value^2^
Age at first pregnancy				0.90
18–29	879 (53%)	447 (53%)	432 (53%)	
30 or more	770 (47%)	394 (47%)	376 (47%)	
Women’s education				0·91
Elementary school	247 (15%)	124 (15%)	123 (15%)	
High school	586 (36%)	302 (36%)	284 (35%)	
Illiterate	408 (25%)	212 (25%)	196 (24%)	
Middle school	408 (25%)	203 (24%)	205 (25%)	
Husband’s education				0·018
Elementary school	252 (15%)	127 (15%)	125 (15%)	
Illiterate	655 (40%)	318 (38%)	337 (42%)	
Middle school	350 (21%)	169 (20%)	181 (22%)	
High school	392 (24%)	227 (27%)	165 (20%)	
Women’s job				0·40
Others	200 (12%)	100 (12%)	100 (12%)	
Worker	34 (2·1%)	13 (1·5%)	21 (2·6%)	
Employee	237 (14%)	127 (15%)	110 (14%)	
Housewife	1 178 (71%)	601 (71%)	577 (71%)	
Husband’s job				0·077
Others	605 (37%)	295 (35%)	310 (38%)	
Worker	652 (40%)	355 (42%)	297 (37%)	
Employee	392 (24%)	191 (23%)	201 (25%)	
Residence type				0·010
Apartment	741 (45%)	404 (48%)	337 (42%)	
House (with yard)	908 (55%)	437 (52%)	471 (58%)	
Vitamin D at baseline				
Normal	217 (13%)	92 (11%)	125 (15%)	0.004
Moderate	744 (45%)	371 (44%)	373 (46%)	
Severe	688 (42%)	378 (45%)	310 (38%)	
Vitamin D at delivery				< 0·001
Normal	543 (33%)	446 (53%)	97 (12%)	
Moderate	729 (44%)	382 (45%)	347 (43%)	
Severe	377 (23%)	13 (1·5%)	364 (45%)	
Birth weight				0·001
Low birth weigh	135 (8·2%)	49 (5·8%)	86 (11%)	
Macrosomia	31 (1·9%)	19 (2·3%)	12 (1·5%)	
Normal	1 483 (90%)	773 (92%)	710 (88%)	
Type of delivery				< 0·001
Caesarean section	539 (33%)	240 (29%)	299 (37%)	
Normal vaginal delivery	1 110 (67%)	601 (71%)	509 (63%)	
Reason of caesarean section				0·002
Fetal distress	23 (1.4%)	7 (0·8%)	16 (2·0%)	
Cephalopelvic disproportion (CPD)	63 (3·8%)	29 (3·4%)	34 (4·2%)	
Prolonged labor	240 (15%)	101 (12%)	139 (17%)	
Malpresentation	157 (9·5%)	82 (9·8%)	75 (9·3%)	
Other reasons	52 (3·2%)	21 (2·5%)	31 (3·8%)	
Preeclampsia (%)	211 (13%)	75 (8·9%)	136 (17%)	< 0·001
Gestational diabetes mellitus	82 (5·0%)	32 (3·8%)	50 (6·2%)	0·026
Preterm delivery (%)	197 (11.9)	76 (9.04)	121 (14.98)	< 0.001

^1^n (%).

^2^Pearson's Chi-squared test.

**Figure 2:**
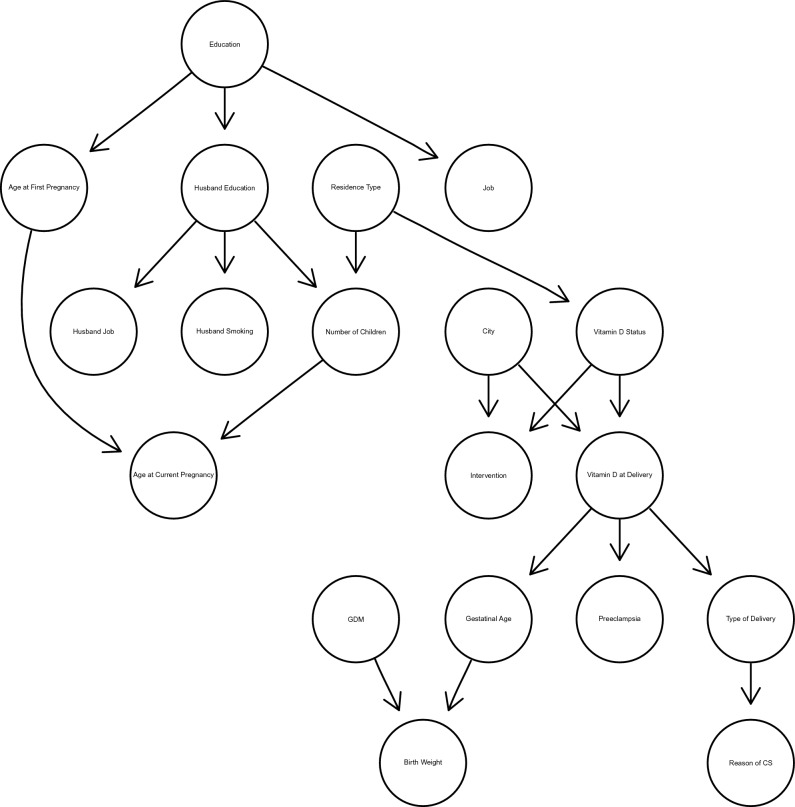
Directed acyclic graph (DAG) generated by the HC structure learning algorithm for the study.

Figure 2 presents the DAG of the study and shows the relationships between the socioeconomic characteristics of participants. The education level of participants was the primary variable that differentiates their living conditions. It directly affects the job status, husband’s education, and age of the first pregnancy of women. The age of the first pregnancy and the number of children determined the age of mothers during the current pregnancy. Among these variables, the residence type (living in a house or apartment) as a determinant of economic level and exposure to the sun was identified as a critical variable affecting the vitamin D status of females.

Vitamin D level at delivery was a function of vitamin D level at the start of the study (baseline) and the city (intervention city vs control city) where females lived. This conditional relationship adjusts the results for baseline status and receiving interventions. The vitamin D level at delivery was related to the mode of delivery, preeclampsia, and gestational age. Finally, the gestational age and GDM determined the birth weight of newborns.

**Figure 3:**
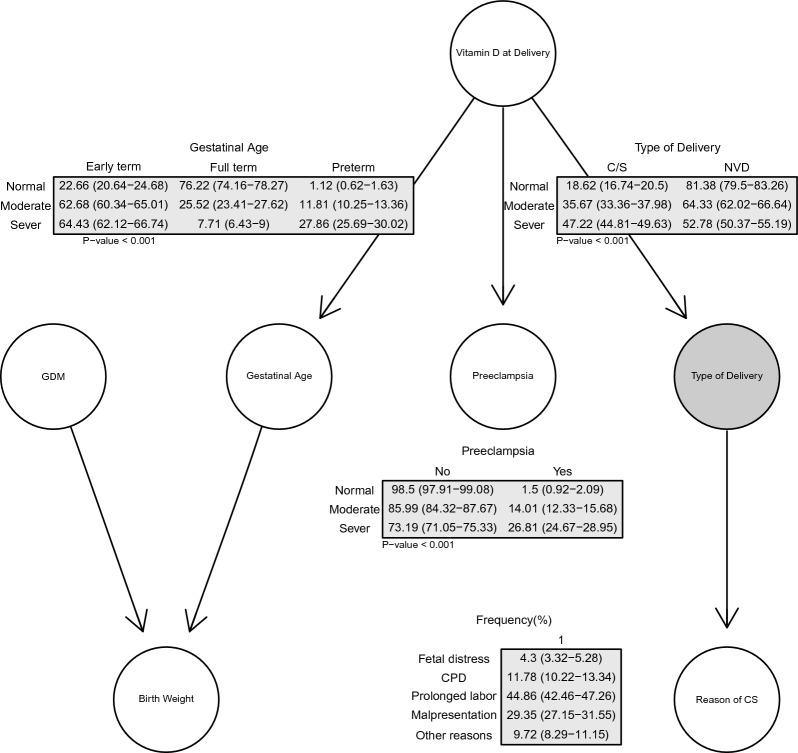
Conditional distribution tables for variables in the BN subset focused on exposure and outcomes.

Conditional probabilities in BN revealed that knowledge about vitamin D level at delivery informed us about the pregnancy outcomes and type of delivery. The type of delivery was conditionally independent of vitamin D status at baseline, given the vitamin D status at the time of delivery. Therefore, in Fig. 3, we focused on the intervention and outcome variables and presented the conditional distribution tables of these variables. According to these tables, the probability of cesarean section increased by the level of vitamin D deficiency from 18.62% (95% CI: 16.74–20.5) at a normal level to 47.22% (95% CI: 44.81–49.63) at severe deficiency levels. The same pattern was observed in an increased risk of preeclampsia and preterm birth at severe and moderate levels of vitamin D deficiency compared with normal status. All the relationships were significant, with *P*-values < 0.001. The most frequent causes of cesarean section were prolonged labor 44.86 (95% CI: 42.46–47.26), malpresentation 29.35 (95% CI: 27.15–31.55), and CPD 11.78% (95% CI: 10.22–13.34).

Four different logistic models were fitted in Table 2. In the first model, one of the direct parents of the outcome (vitamin D at delivery) was added. In the second model, the second-order ancestors (vitamin D status at the beginning of the study and city) were added. In the third model, the third-order ancestor (residence type) was added, and finally, the female's education level and age at the current pregnancy were added as potential confounders. Based on our findings, there was a negative association between moderate and severe vitamin D deficiency at the time of delivery and the occurrence of NVD. The OR in model 1 implies to 60% and 69% reduction in the odds of NVD for moderate and severe deficiency, respectively. The adjusted ORs in other models were almost the same and showed nearly 71% and 77% reduction in the odds of NVD.

**Table 2:** Unadjusted and adjusted logistic regression models to estimate the odds ratio (OR) of cesarean section in participants of the study.

Characteristic	Model 1*			Model 2			Model 3			Model 4		
	OR^1^	95% CI^1^	*P* value	OR^1^	95% CI^1^	*P* value	OR^1^	95% CI^1^	*P* value	OR^1^	95% CI^1^	*P* value
Vitamin D at delivery (Reference: normal)												
Moderate	0.40	0.29, 0.54	< 0.001	0.29	0.20, 0.41	< 0.001	0.29	0.21, 0.41	< 0.001	0.28	0.19, 0.39	< 0.001
Sever	0.31	0.22, 0.44	< 0.001	0.22	0.13, 0.35	< 0.001	0.23	0.14, 0.38	< 0.001	0.21	0.12, 0.34	< 0.001
Vitamin D at baseline (Reference: normal > 20)												
Moderate 10–20				2.51	1.65, 3.83	< 0.001	2.73	1.79, 4.19	< 0.001	2.68	1.75, 4.13	< 0.001
Severe < 10				1.56	1.01, 2.44	0.049	1.96	1.22, 3.16	0.006	1.82	1.12, 2.96	0.015
Intervention with vitamin D supplementation (reference: control)												
Intervention				1.31	0.94, 1.82	0.11	1.25	0.90, 1.75	0.2	1.33	0.95, 1.86	0.10
Residence type (Base = apartment)												
House							1.42	1.06, 1.90	0.021	1.12	0.81, 1.54	0.5
Education (Reference: Elementary school)												
High school										0.74	0.47, 1.15	0.2
Illiterate										0.56	0.35, 0.89	0.015
Middle school										0.92	0.58, 1.46	0.7
Age at current pregnancy (Reference: 18–29)												
More than 30										1.47	1.11, 1.93	0.006
Sensitivity		39.22			35.95			28.76			38.56	
Specificity		84.80			46.32			78.94			78.07	
Accuracy		70.71			63.84			63.43			65.86	

^1^OR = Odds Ratio, CI = Confidence Interval, *Model 1 is adjusted only for a direct parent of CS (Vitamin D at delivery), Model 2 added the second order parents (Baseline vitamin D status & Intervention with vitamin D supplementation), Model 3 added the third order parents (Residence type), finally, model 4 added all other ancestors (Education, Age at current pregnancy).

**Figure 4:**
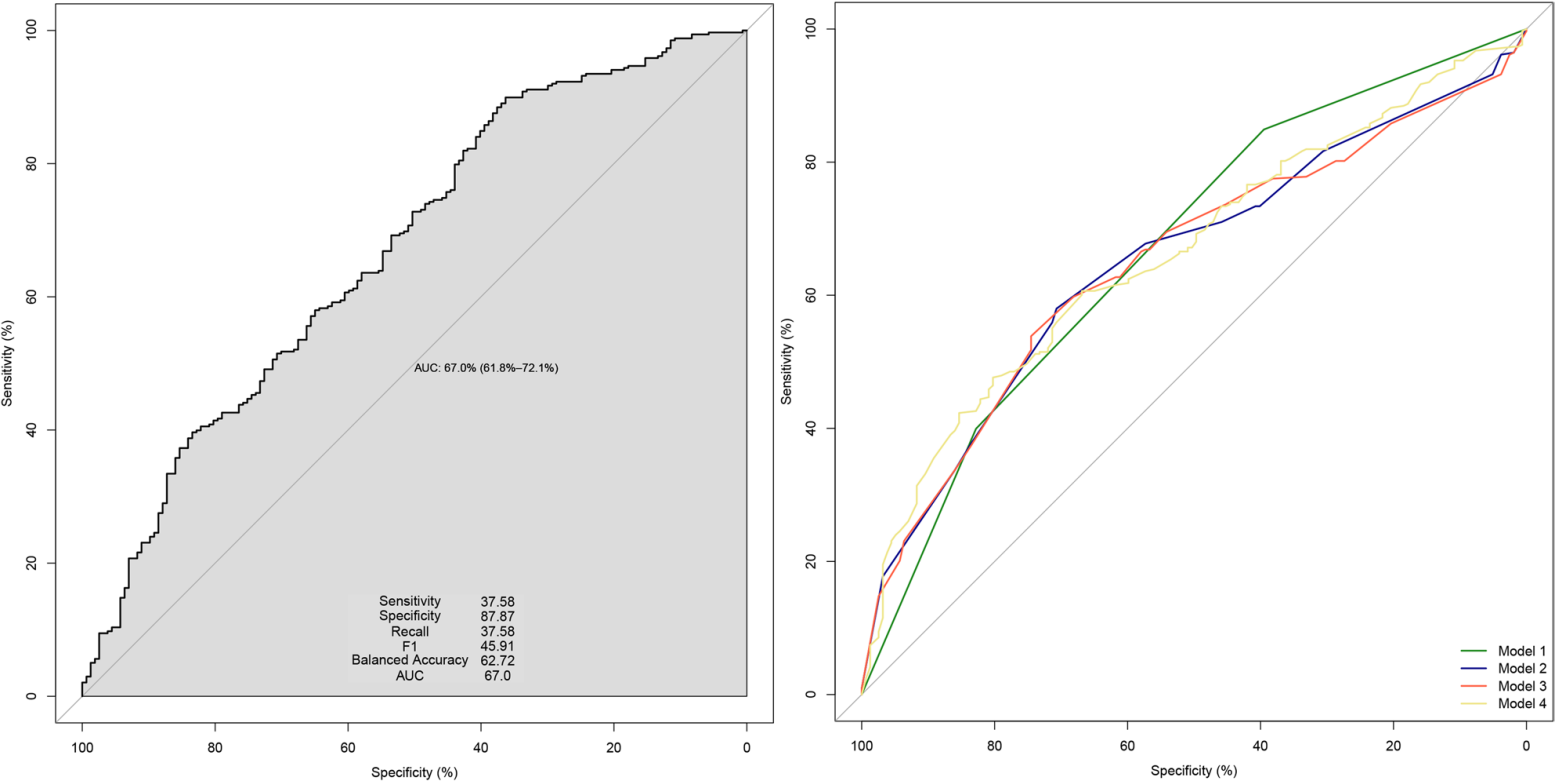
Receiver operating characteristic (ROC) curve comparing BN and ordinary logistic regression models.

Model 1 had an AUC of 67% (95% CI: 62.2–71.7), with a sensitivity of 39.22%, specificity of 8.82%, and accuracy of 70.71%. These metrics suggest superior performance compared to the other models in Table 2. The ROC curves of all these models and the BN are presented in Fig. 4. The AUC of BN in the left-hand panel is almost similar to model 1, and the AUC is the same. The sensitivity and specificity of correct prediction for participants with cesarean section in our BN are 37.58% and 87.87%, respectively, leading to 62.72% accuracy.

## Discussion

In the present study, we explored various influential factors related to the association between serum concentrations of 25(OH)D at delivery and mode of delivery using a BN approach. This method has several advantages, including the ability to manage missing data and collinear data effectively^41,42^ as well as the capacity to handle complex problems with uncertainties and associations^18,43^. Based on our investigation, we report a significant association between vitamin D levels and the mode of delivery. Specifically, our findings indicate that women with moderate and severe vitamin D deficiency had increased odds of undergoing cesarean section compared to those with normal levels of this essential nutrient. Furthermore, our analysis revealed an indirect effect of vitamin D status on pregnancy outcomes, as serum concentrations of 25(OH)D at delivery were positively correlated with the incidence of preeclampsia and gestational age. Importantly, we identified two critical factors that impact 25(OH)D values at delivery: baseline vitamin D concentrations and receiving vitamin D supplementation during pregnancy. Additionally, we found that the type of residence (house vs apartment) was associated with differences in vitamin D levels. Taken together, our results demonstrate the importance of monitoring and maintaining optimal vitamin D levels during pregnancy for optimal maternal and fetal health outcomes.

Previous studies have evaluated the relationship between maternal vitamin D status and mode of delivery using various statistical methods, yielding inconsistent findings^12–17,29–32^. Many studies have utilized regression models with and/or without adjustment for potential confounding variables, documenting an increased incidence of cesarean section in mothers with vitamin D deficiency^12–17^. For example, after controlling for factors such as race, age, education level, insurance status, and alcohol use, a cross-sectional study on 253 American women revealed that participants with 25(OH)D levels less than 37.5 nmol/liter had almost four times the odds of cesarean delivery compared to those with levels of 25(OH)D 37.5 nmol/liter or greater^12^. Another cross-sectional study on 200 Indian pregnant women with singleton pregnancies showed a higher prevalence of hypovitaminosis D among mothers who underwent cesarean delivery compared to those who delivered vaginally (92% of women with vitamin D deficiency and 6% with insufficiency versus 85.6% with deficiency and 10.8% with insufficiency)^14^. A case–control study of 60 Danish women demonstrated a significant association between serum 25(OH)D levels and an increased risk of emergency cesarean section due to dystocia, highlighting the importance of vitamin D in the preparation of the uterus muscles and smooth muscle contractility during normal vaginal delivery^44^. Similarly, another study on 2,251 low-income pregnancies demonstrated that after adjusting for energy intake, other nutrients, and other potentially confounding variables, women with vitamin D deficiency had a two-fold increased risk of cesarean delivery due to prolonged labor compared to those with sufficient serum concentrations^45^. Additionally, a cross-sectional study of Iranian women with singleton pregnancies aged 15–45 years suggested that vitamin D deficiency was associated with a higher rate of cesarean delivery and other adverse maternal and neonatal outcomes^13^. Data from a multi-ethnic Asian cohort study of 940 women showed that Chinese and Indian women with 25 (OH) D inadequacy had higher odds ratios of emergency cesarean Sect. (1.9 and 2.41-fold, respectively) than those with sufficient values. Although this study reported an inverse association between maternal 25 (OH)D concentrations and fasting plasma glucose, there was no association between vitamin D insufficiency and risk of GDM in the overall cohort or within any ethnic group^16^. A prospective study of 995 singleton pregnancies found that after adjusting for maternal age, BMI, racial origin, smoking, method of conception and season of blood testing, first-trimester maternal serum levels of 25(OH)D were similar between women who delivered vaginally and those required elective or emergency caesarean delivery^29^. However, it is important to note that although levels of 25(OH)D in the first trimester were similar among all participants, those with declining vitamin levels during pregnancy may have a higher risk of cesarean delivery than those who maintain their vitamin D levels more effectively. Data from a prospective cohort study of 1153 low-income and minority gravidae revealed that after adjustment for age, parity, ethnicity, smoking, BMI, gestation, and season at entry, the cesarean delivery risk was significantly increased for women with vitamin D deficiency, but not for those with vitamin D insufficiency. The authors found that vitamin D deficiency was associated with a two-fold increased risk of cesarean delivery due to prolonged labor^17^. However, some investigations report no significant association between 25(OH)D and mode of delivery^29–32^. For example, a study on 461 Nigerian pregnant women found no significant differences between study groups based on 25(OH)D levels (normal, insufficient, and deficient) regarding the rate of cesarean delivery or other pregnancy complications such as preeclampsia, GDM, and preterm delivery^30^. Similarly, a cross-sectional study of 154 Turkish pregnant women showed no significant difference in the rate of cesarean delivery between women with serum vitamin D levels < 15 ng/ml compared to those with values > 15 ng/ml^31^. Conflicting results among studies could be attributed to limitations such as small sample size, lack of adjustment for potential confounders, and measurement of vitamin D in the first trimester.

The mechanisms underlying the association between 25(OH)D deficiency and an increased risk of cesarean section are complex and require further investigation. One potential explanation for this association is the presence of vitamin D receptors in skeletal muscle^46^, which can lead to proximal muscle weakness^47^. and suboptimal muscle function and strength in individuals with vitamin D deficiency^3,47^. Additionally, calcium levels, which are regulated by 25(OH)D, play a critical role in smooth muscle performance during labor initiation^18^. Adequate levels of serum calcium are necessary for initiating labor, and vitamin D deficiency may negatively impact both skeletal muscle and smooth muscle strength, leading to specific etiologies of cesarean delivery such as CPD or failure to progress^12^. Vitamin D may affect myometrial contractility via two pathways: involving the intracellular vitamin D receptor and changes in the calcium metabolism^44^. Invitro studies have demonstrated that vitamin D regulates contractile proteins in myometrial cells^48^. Our analysis predicted that 44.86% of pregnant women will undergo cesarean section due to prolonged labor. Thus, maternal vitamin D deficiency at delivery might increase the probability of cesarean section by reducing the ability to push and more complicated and extended labor.

Moreover, maternal vitamin D deficiency during pregnancy and at delivery has been implicated in the pathogenesis of some maternal medical conditions, including preeclampsia, GDM, and preterm delivery, which can be associated with a higher risk of cesarean Sect.^8,9^. A recent systematic review and meta-analysis reported a strong link between maternal vitamin D deficiency and severe preeclampsia, and vitamin D supplementation may be beneficial in preventing this pregnancy complication^49^. Vitamin D deficiency is also associated with many risk factors for endothelial dysfunction and vascular health impairment^19^. On the other hand, adequate vitamin D intake can maintain calcium homeostasis, which is inversely correlated with blood pressure^50^ and may directly suppress the proliferation of vascular smooth muscle cells. Vitamin D may also play a critical role in blood pressure control by regulating the renin-angiotensin system^51^. Additionally, vitamin D can modulate the synthesis of adipokines related to endothelial and vascular health^52^. Vitamin D concentration can affect glucose homeostasis via various mechanisms, such as improving the insulin sensitivity of target cells (liver, skeletal muscle and adipose tissue), improving and protecting β-cell function, and reducing insulin resistant through immunoregulatory and anti-inflammatory effects^20,21^. Women with vitamin D deficiency might be at risk for GDM^22^. Moreover, vitamin D can influence the pathophysiology of preterm delivery through inflammation and immunomodulation processes; pregnant women with vitamin D deficiency might be susceptible to infection, which is an important predisposing factor for preterm delivery^10^. A meta-analysis of 10 studies including 10,098 participants showed that maternal serum concentrations of 25(OH)D levels less than 20 ng/mL were significantly associated with an increased risk of preterm delivery^53^. Our study based on the structure of BN revealed that moderate and severe vitamin D deficiencies at delivery significantly increase the probability of pregnancy complications like cesarean section, preeclampsia, and preterm delivery, but not GDM. Nevertheless, the absence of a significant relationship between 25(OH)D status and GDM in our study could be due to the conditionally dependent approach of the model. Therefore, no significant relationship between vitamin D concentrations and GDM given the city does not necessarily imply that the relationship does not exist overall. Our findings indicated that fetal distress contributed to approximately 4.3% of cesarean section cases. Fetal distress can partly reflect maternal medical conditions, such as preeclampsia, gestational diabetes, and preterm delivery, which are indirectly associated with cesarean Sect.^23–25^. Additionally, previous studies have demonstrated that GDM can increase fetal weight and macrosomia, leading to an elevated risk of elective cesarean section and emergency cesarean due to CPD and dystocia^28^.

We utilized logistic regression models, the most commonly used statistical approach in medical research, to estimate the OR of cesarean section in women with maternal vitamin D deficiency after adjusting confounders. Our results from logistic regression provided similar to those obtained from the BN. In particular, our unadjusted logistic regression model (model 1) showed higher OR for cesarean section in pregnant women with moderate and severe vitamin D deficiency compared with those with normal vitamin D levels. This finding remained significant even after adjusting for other factors such as vitamin D at baseline and city (intervention with vitamin D supplementation) (model 2), vitamin D at baseline, city, and residence type (model 3), and vitamin D at baseline, city, residence type, education, and age at current pregnancy (model 4). Additionally, we found that the AUC of all these models was comparable to that of the BN approach.

To the best of our knowledge, this is the first population-based study globally to illustrate the relationship between the association between 25(OH)D concentrations at delivery and mode of delivery using both BN and logistic regression models. Our study has several strengths, including its population-based design, measurement of 25(OH)D at both baseline and delivery, and the use of appropriate statistical methods for data analysis. These methods have been shown to be superior to traditional regression analysis in various fields of epidemiological studies^18,41–43^.

However, several limitations should be kept in mind when interpreting our findings. Firstly, we could not apply the liquid chromatography technique as the gold standard method for measuring the circulating levels of 25(OH)D, due to inaccessibility. Nevertheless, the ELISA technique is reliable when performed by expert staff^54^. Secondly, there were variations in vitamin D supplementation intake among the study population. However, our results suggest that vitamin D was the critical determinant of the mode of delivery, regardless of vitamin D at baseline and intervention. Additionally, we had no data on UVB exposure during pregnancy trimesters, calcium intake, sartorial habits, and skin types. Thirdly, our study population was limited to two cities mainly composed of the Persian ethnic group, thus generalizing our results to other ethnicities should be approached with caution. Fourthly, we could not measure myometrial dysfunction by contraction frequency or Montevideo units due to a lack of data. Finally, since the cesarean rate is very high in many countries, including Iran, our findings may not be generalizable to populations with a lower cesarean rate.

In conclusion, our study highlights the adverse effects of maternal vitamin D deficiency on mode of delivery, both directly and indirectly, through maternal complications such as preeclampsia and preterm delivery, leading to a higher probability of cesarean section. Therefore, screening all pregnant women for serum concentrations of 25(OH)D can help identify mothers at risk of adverse pregnancy outcomes.

## Data Availability

The datasets generated during the current study are available from the corresponding author upon reasonable request.
